# Study on effectiveness of transfusion program in thalassemia major patients receiving multiple blood transfusions at a transfusion centre in Western India

**DOI:** 10.4103/0973-6247.67029

**Published:** 2010-07

**Authors:** Neeraj Shah, Anupa Mishra, Dhaval Chauhan, C. Vora, N. R. Shah

**Affiliations:** *MBBS Student (Intern Doctor), Smt. NHL Municipal Medical College, Ahmedabad, India*; 1 *Medical Director, Indian Red Cross Society (IRCS), Ahmedabad, India*; 2 *Department of Pathology, Smt. NHL Municipal Medical College, Ahmedabad, India*

**Keywords:** Beta-thalassemia major, chelation, HCV positivity, iron overload

## Abstract

**Background:**

Children suffering from beta-thalassemia major require repeated blood transfusions which may be associated with dangers like iron overload and contraction of infections such as HIV, HCV, and HBsAg which ultimately curtail their life span. On the other hand, inadequate transfusions lead to severe anemia and general fatigue and debility.

**Materials and Methods:**

Data were obtained from 142 beta-thalassemia major patients aged 3 years or more receiving regular blood transfusions at a transfusion centre in Western India from 1 April 2009 to 30 June 2009. The clinical data and laboratory results were subsequently analyzed.

**Results:**

Of the 142 patients, 76 (53.5%) were undertransfused (mean Hb <10 gm%). 96 (67%) of the patients were taking some form of chelation therapy but out of them only 2 (2%) were adequately chelated (S. ferritin <1000 ng/ml). 5 (3.5%) of the patients were known diabetics on insulin therapy. 103 (72%) of the patients were retarded in terms of growth. The prevalence of transfusion-transmitted infections (TTIs) such as HCV, HIV, and HBsAg was respectively 45%, 2%, and 2%, with the prevalence of HCV being significantly more than the general population. The HCV prevalence showed positive correlation with the age of the patients and with the total no of blood transfusions received. As many as 15% (6 out of 40) children who were born on or after 2002 were HCV positive despite the blood they received being subjected to screening for HCV.

**Conclusions:**

The study suggests the need to step up the transfusions to achieve hemoglobin goal of 10 gm% (as per the moderate transfusion regimen) and also to institute urgent and effective chelation measures with the aim of keeping serum ferritin levels below 1000 ng/ml to avoid the systemic effects of iron overload. In addition, strict monitoring of the children for endocrinopathy and other systemic effects of iron overload should be done. Rigid implementation of quality control measures for the ELISA kits used to detect HCV in donor blood needs to be done urgently. Alternately, more sensitive and specific measures (like NAT testing) should be employed for detection of HCV. In the absence of a definitive cure accessible and available to all patients, strict implementation of the above suggested measures will go a long way in improving the quality (and quantity) of life in patients of beta-thalassemia major.

## Introduction

Beta-thalassemia major is a transfusion-dependent severe anemia requiring lifelong blood transfusions for the afflicted patients to stay alive. It is also a major health problem in Gujarat State, India (prevalence 7.48%[1]) especially in some communities such as Sindhis, Muslims, etc. Thalassemic children experience various problems if the transfusion is inadequate but at the same time repeated blood transfusions are associated with hazards like iron overload and risk of acquiring transfusion-transmitted infections (TTIs). Iron overload can lead to endocrinal dysfunction in the form of growth retardation and diabetes mellitus. TTIs such as HIV (with risk of progession to AIDS), HBsAg, and HCV (with high risk of developing chronic hepatitis, liver cirrhosis and hepatocellular carcinoma) can also occur. Thus, chronic blood transfusion in thalassemic patients is a double-edged sword. Ultimately thalassemic patients die either due to transfusions or due to lack of it, with the result that they seldom survive beyond the age of 25 years.

## Materials and Methods

The study was conducted among 142 beta-thalassemia major patients receiving regular blood transfusions at the Indian Red Cross Society (IRCS), Ahmedabad branch located at Ahmedabad City, Gujarat state, India, from 1^st^ April 2009 to 30^th^ June 2009.

All confirmed beta-thalassemia major patients aged more than or equal to 3 years registered at IRCS, Ahmedabad, and receiving blood transfusions regularly at the same institute were included in this study. After obtaining due permission from the authorities, the clinical data were collected by detailed interviewing of the patients and/or the relatives present through preformed questionnaires. In addition, detailed clinical examination, including anthropometric measurements and other clinical parameters relevant to the project, was also carried out. Blood of the patients who had not been tested for their HIV, HBsAg, and HCV status as well as serum ferritin levels over the past 1 year, was sent for the same. The data thus obtained, including the laboratory results and the clinical examination results, were subsequently analyzed.

## Results

Our study comprised 142 beta-thalassemia major patients with 54 (38%) females and 88 (62%) males receiving regular blood transfusions at the Indian Red Cross Society, Ahmedabad branch, youngest patient is 3 years old and the oldest is 26 years with the mean age being 12 years.

### Religion and Consanguinity

As seen in Table 1 Muslims and Sindhis who although form a minority among the Indian population form a major chunk among thalassemic patients with 20% (28/142) of them being Muslims and 16% (23/142) Sindhis. In coherence with the inheritance pattern of autosomal recessive diseases (beta-thalassemia major being one), consanguinity was found in a significant proportion i.e. 17/142 or 12% of the parents of the patients. Muslim community, hereto known for such marital practices, was found to be the major contributor with 15/17 consanguineous marriages having taken place amongst parents of Muslim patients. It was a striking revelation that 53% (15/28) of the Muslim patients were born out of a consanguineous marriage.

**Table 1:** Relation between religion and consanguineous marriage in parents of thalassemia major patients

		Consanguinity		Total
		No	Yes	
Religion	Hindu	91	0	91
	Muslim	13	15	28
	Sindhi	21	2	23
Total		125	17	142

### Adequacy of Transfusions

Considering 10 gm% (in accordance with the moderate transfusion regimen) of pretransfusion hemoglobin as the cut off between adequately transfused and under transfused patients, we found that among those receiving transfusions once a month, 62% were under transfused. Similar percentages for those receiving 2, 3, and 4 transfusions per month were 50%, 43%, and 50%, respectively. Overall 53.5% i.e. 76/142 would do better with increasing the frequency of blood transfusions.

### Relationship between Age and Number of Blood Transfusions:

**Figure 1:**
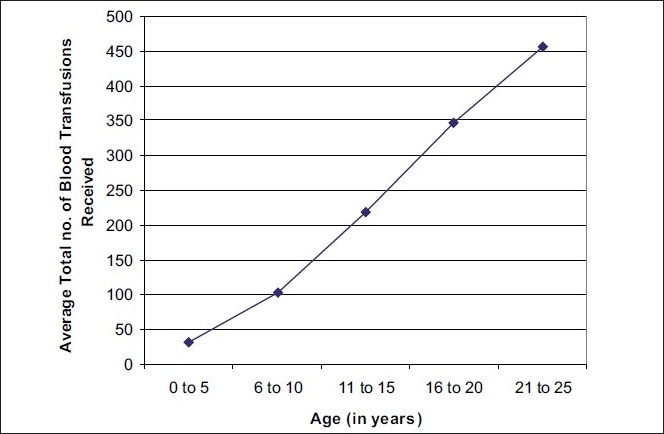
Relation between age and average total no. of blood transfusions received

Figure 1 shows a linear relationship between the age of thalassemia major patients and total number of blood transfusions received so far. With the increase in age, the cumulative number of blood transfusions received will increase, as is depicted in the graph.

**Figure 2:**
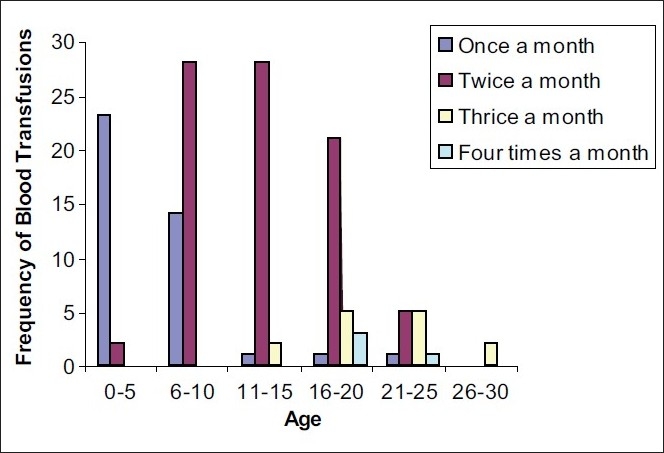
Relation between age and frequency of blood transfusions per month

Figure 2 shows that with the increase in age, frequency of blood transfusions received per month also goes up. Such a relation is expected, as due to worsening of the disease with progression of age, the requirement for blood transfusions will increase. Most of the patients aged 0-5 years receive blood transfusions only once a month, which is adequate considering their age. However, a major chunk of patients aged 11-20 years still receive blood transfusions only twice a month and looking at the pre transfusion hemoglobin values (see above), such a frequency of blood transfusions appears to be inadequate and needs to be reviewed along with consideration for other relevant factors.

### Adequacy of Chelation

Under conditions of ideal chelation it is expected that serum ferritin levels be maintained within normal limits irrespective of the total no. of transfusions. However such a uniform maintenance of serum ferritin levels was not found indicating irregular and inadequate chelation practices and/or variable response to chelation therapy. Setting the cut off limit for S. ferritin <1000 ng/ml between adequately chelated and poorly chelated patients,[2,3] we found that only 9/142 (6.3%) patients had S. ferritin <1000 ng/ml (all those patients were below 10 yrs of age) and seven out of these nine patients were not taking any chelation. Hence only two (1.4%) patients could be considered to be taking adequate chelation therapy. The remaining 133 patients need their chelation regimen to be reviewed. At the time of our study 96 (67%) patients were taking some form of chelation therapy. Out of these 96 patients taking chelation, only 2 (2%) are taking adequate chelation and hence the dose and/or the type of the chelating agent(s) needs to be modified in 94 of the patients already on chelation therapy. Out of the remaining 46 (33%) patients, only 7 have maintained adequate ferritin levels (<1000 ng/ml) without chelation. Hence we need to review those 39 patients who are not taking any chelation therapy at present. Out of these 39 patients, 23 patients had taken chelation at some point of time but had to discontinue it due to either joint pain (13/23) or leucopenia (10/23). However this still leaves us with 16 people who have S. ferritin >1000ng/ml and who have not taken ANY chelation therapy yet.

**Figure 3:**
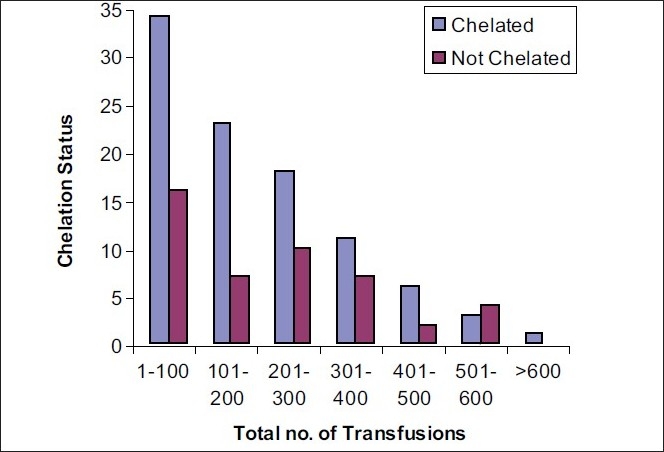
Relation between chelation status and total no. of transfusions received

Figure 3 shows the relative proportion of patients who are receiving and who are not receiving some form of chelation therapy, in terms of the total no. of blood transfusions received so far. For patients who have received <100 transfusions so far, almost a third of them are not on any kind of chelation therapy. This is acceptable as we do not expect serum ferritin levels to be very high in these patients. However, for the patients who have received 201-400 blood transfusions, more than a third of them are NOT on any kind of chelation therapy yet. In the patients who have received 501-600 transfusions, >50% are not on any chelation therapy yet, which is indeed alarming.

### Diabetes and Ferritin

In our study there are 5/142 (3.5%) known diabetic patients and all of them had ferritin levels above 6000 ng/ml. Their ages in descending order were 25,24,20,18, and 13 years and all the patients are on insulin therapy. This is in accord with the fact that diabetes in these patients is usually due to iron deposition in the pancreas leading to insulin deficiency. We have not performed blood sugar testing of all the 142 patients hence we could not detect latent cases of diabetes, if any.

### Growth Retardation

Out of the 142 patients, 103 (72%) were below 2 standard deviations of ICMR 1990 standards of weight for age (<80% of W/A) and 76 (53%) were 2 SD below ICMR 1990 standards of height for age (<90% of H/A). While 63 (44%) were lagging behind in both W/A and H/A i.e. they were both wasted and stunted. Thus a high proportion of the patients show growth retardation, which may be due to iron deposition in the pituitary gland: another consequence of inadequate chelation and high ferritin levels.

### Prevalence of Transfusion Transmitted Infections (TTIs)

In our study group of 142 pts, we studied the prevalence of three important TTIs (HIV, HBV and HCV) which is compared to the prevalence of these infections in voluntary blood donors (taken as indicative of general population) shown in Table 2.

**Table 2:** Comparison of prevalence of HCV, HBsAg and HIV in thalassemia major patients and voluntary blood donors

TTIs	No. (out of 142)	% positivity	Voluntary blood donor prevalence[4]	*P* values
HCV positive	64	45	Less than 2%[5]	<0.001
HBsAg positive	3	2	1.19%[4,6]	>0.05
HIV positive	3	2	0.36% in 2006 (NACO data)	>0.05

A striking 45% of the patients are HCV positive which is significantly more than the prevalence in general population (*P*<0.001). The prevalence of HIV positivity is not statistically significant compared to the general population. However, the data available from NACO and voluntary blood donor studies include only adolescents and adults while our study includes thalassemic patients majority of whom are under 18 years of age. The three HIV positive patients in the study are aged 8, 11 and 12 years. There is no significant difference in HbsAg positivity in the thalassemic patients and general population (the ages of the HbsAg positive children are 3, 4, and 8 years, respectively). All the 3 patients who are HIV positive are HCV positive as well. One patient is unfortunate to be HCV, HIV as well as HBsAg positive.

Since a large proportion of patients are HCV positive, we have analyzed our data by finding correlation between:

#### (1) HCV positivity and age

**Figure 4:**
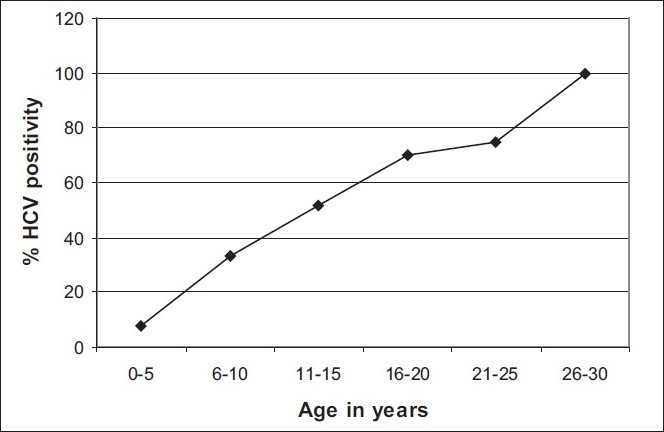
Correlation between HCV positivity and age (in years) of thalassemia major patients

With age the number of blood transfusions received increases and so does the risk of acquiring TTIs. This is shown in Figure 4 by a linear relationship between age and HCV positivity which indicates that the more the age of the patient the more is the chance of him/her being HCV positive.

#### (2) HCV positivity of total no. of BTs

**Figure 5:**
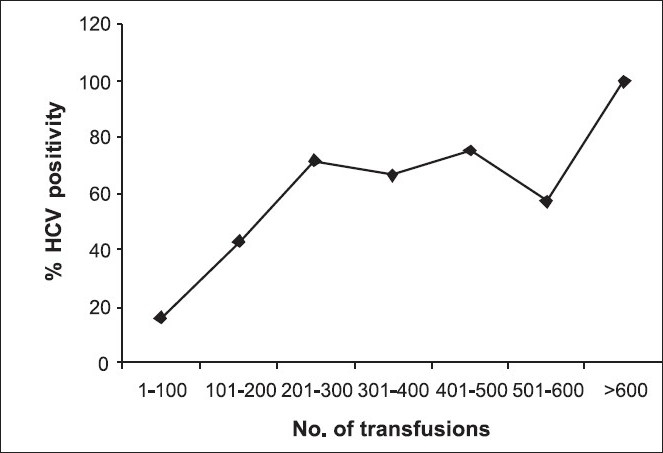
Correlation between HCV positivity and total no. of blood transfusions received till date by thalassemia major patients

As seen in Figure 5, except for unexplained dips at 301-400 and 501-600 transfusions, a linear relationship is found between HCV positivity and total number of blood transfusions received so far.

In India mandatory screening for HCV was introduced in 2002. It therefore follows that thalassemic children born in 2002 or later should not ideally be HCV positive. However, out of the 64 patients who are HCV positive, 6 patients (6/142 or 4.2%) are below 8 years of age. In all these HCV-positive cases the causal role of blood transfusion can only be logically established by documenting the absence of HCV infection in mothers before or during pregnancy. There are total 40 children who are below 8 years of age and out of them 6 children i.e. 6/40 or 15% are HCV positive despite the fact that the blood they received was subjected to screening for HCV.

## Discussion

Beta-thalassemia major is one of major public health problems in India. The general incidence of thalassemia trait in India varies between 3 and 17%.[7] It is estimated that there are about 65,000-67,000 beta-thalassemia patients in India with around 9,000-10,000 cases being added every year.[8] The overall prevalence of beta thalassemia major in Gujarat State, Western India, is 7.48%.[1]

As 76 (53.5%) of the patients are under transfused, the moderate transfusion regimen (hemoglobin goal of 10 gm%) being practiced at this centre requires proper and more meticulous implementation. The moderate transfusion regimen practiced correctly has been shown to ensure normal growth without excessive expansion of erythropoiesis, and with effective prevention of iron overload.[9] The inevitable consequence of regular life-saving transfusions in thalassemia major is the accumulation of excess iron within tissues. This causes progressive organ damage and dysfunction which, without treatment, can lead to an increase in morbidity and mortality.[10] For patients requiring regular blood transfusions, iron chelation may represent life-saving therapy. A landmark study investigating role of desferoxamine (Desferal^®^) in prevention of complications of transfusional iron overload showed that survival to at least 25 years of age in poorly chelated β-thalassemia major patients was just one-third that of patients whose iron levels were well managed by deferoxamine.[11] The optimal age for initiating iron chelation therapy in patients with severe thalassemia remains uncertain, although in theory it should begin as early as possible to prevent growth and developmental defects. Guidelines from the Thalassemia International Federation recommend that chelation therapy is initiated when serum ferritin levels reach approximately 1000 ng/mL, which usually occurs after the first 10 to 20 transfusions or around 2-3 years of age.[2,3]

In the present study, we found that only 9/142 (6.3%) patients had S. ferritin <1000 ng/ml and 7 out of these 9 patients were not taking any chelation. Hence only 2 (1.4%) patients could be considered to be taking adequate chelation therapy. 96 (67%) patients were taking some form of chelation therapy. Out of these, 89 patients were taking Kelfer (Deferiprone), 5 were taking Asunra (Desferasirox), and 2 were taking Desferral (Desferrioxamine). 23 patients had to discontinue Kelfer either due to joint pain (13/23) or leucopenia (10/23). Three approved iron chelation agents are available currently---deferasirox, deferoxamine, and deferiprone. The agent used most commonly is oral deferiprone (Kelfer) and its side effects are joint pain, leucopenia, and GI intolerance (nausea, vomiting). Desferrioxamine (Desferal) is to be administered subcutaneously and hence is inconvenient for the patients and it is used only when patient is sensitive to deferiprone or deferiprone alone is ineffective. Desferasirox (Asunra) is a newer agent. It is also given orally but is expensive.

Iron-overload-associated endocrinopathy is the most frequently reported complication of chronic transfusion therapy in patients with thalassaemia. In a study carried out to determine role of hemoglobinopathy in development of endocrinopathy, the patients with transfusion-dependant thalassemia, transfusion-dependant sickle cell disease, and non-transfusion-dependant sickle cell disease (SCD) were studied for prevalence of endocrinopathy. Subjects with thalassemia had more evidence of diabetes, hypogonadism, hypothyroidism, and growth failure, versus transfusion-dependent SCD (sickle cell disease).[12] 56% of thalassemics had more than one endocrinopathy compared with only 13% of patients having SCD (*P* < 0.001). In contrast, transfusion-dependent SCD was not different from non-transfusion-dependent SCD (sickle cell disease). Multivariate analysis indicated that endocrinopathy was more likely in thalassemics than in SCD (sickle cell disease) [Odds Ratio (OR) = 9.4, *P* < 0.001], with duration of chronic transfusion a significant predictor.[12] In our study there are 5/142 (3.5%) known diabetic patients and all of them had ferritin levels above 6000 ng/ml. High serum ferritin levels during puberty are a risk factor for hypogonadism, and high serum ferritin levels during the first decade of life predict final short stature.[13] It remains to be determined whether improving chelation by earlier initiation of desferioxamine or by the combined use of desferioxamine and deferiprone will lead to better growth and sexual development without desferioxamine toxicity. In the present study, 72% of children were wasted, 53% were stunted, and 44% were both wasted and stunted according to ICMR 1990 standards of weight for age and height for age.

In India, mandatory screening for HCV was introduced as late as 2002. The prevalence of HCV was found to be as high as 21% in thalassemia patients and correlated with advancing age, indicative that they may have acquired it in the period when screening of blood units for HCV was not mandatory.[14] In two studies from Western India, the prevalence of HCV in multiple transfused thalasemics was 16.7% and 17.5%, respectively.[5,6] In our study the prevalence of HCV, HIV, and HbsAg was respectively 45%, 2%, and 2%. These findings when compared with the prevalence data in voluntary blood donors prove that the prevalence rate for HCV is statistically very significant. It is required to collect data about the HCV positivity of mothers of six patients (out of a total of 64 HCV positive patients), aged less than 8 years, so as to ascribe the real reason(s) for HCV positivity in them.

In summary, the effectiveness of the transfusion program is evaluated on the following counts:

- The frequency of blood transfusions given per month appears to be inadequate especially for patients aged 11-20 years of age.
- In terms of pre transfusion hemoglobin levels (taking 10 gm% as cut off), 53.5% of patients are undertransfused.
- In terms of serum ferritin levels (taking 1000 ng/ml as cut off), nearly 93% of patients need their chelation regime to be reviewed.
- In terms of the total number of blood transfusions received, more than a third of all patients who have received >200 blood transfusions are still not on chelation therapy. Thus, the chelation regime is not appropriate.
- In terms of the prevalence of endocrinopahies like diabetes mellitus and growth retardation, the chelation therapy received by the patients is inadequate.
- Looking at the high prevalence of HCV, screening of the blood units for TTIs especially HCV needs to be improved.
- For the ultimate effectiveness of transfusion regime, other factors like splenic sequestration, presence of alloantibodies as well as quality aspects of red cell concentrates also need to be studied and taken care of.

## Conclusions

The present study critically evaluated the current transfusion regime, adequacy and regularity of chelation program, the side effects of multiple transfusions in terms of iron accumulation and the resultant damage to various organs because of it, prevalence of three major transfusion-associated infections, namely infections by HIV, HCV, and HBV in thalassemia major patients enrolled at IRCS centre in Western India. 15% prevalence of HCV positivity among children born after 2002 is indeed high in the post screening era. It is suggested that efforts should be made to identify the real reasons for higher prevalence of HCV which might include comparison of ELISA kits of different manufacturers, rigid implementation of quality control measures while testing, and use of more specific and sensitive methods like NAT testing for HCV. Again it is also suggested that the family members’ data regarding positivity of important viral markers is included as an essential part of the records of these patients. It is also suggested to revise and devise suitable transfusion regime so that a balance between adequate transfusion and minimum of side effects of multiple transfusions is maintained. The chelation regimen of 133/142 patients needs to be reviewed. The 16 patients whose serum ferritin is >1000 ng/ml and who have not been put on any chelation regimen till date require to be urgently put on appropriate chelation regime. Systemic effects of multiple transfusions should be rigorously and meticulously studied. We recommend regular screening of all thalassemic patients especially those with high ferritin levels for diabetes mellitus by periodic blood glucose testing. This is the first study of its kind at Indian Red Cross Society (IRCS), Ahmedabad, Western India. Hence, it is suggested to repeat the study after implementation of various measures suggested above so that the ultimate goal of providing safest and appropriate transfusion support to thalassemics is achieved.
